# Multilevel Intervention to Support Tailored and Responsive HIV Pre-Exposure Prophylaxis Care in Rural North Carolina: Protocol for a Randomized Controlled Trial

**DOI:** 10.2196/68085

**Published:** 2025-03-21

**Authors:** Sarah E Rutstein, Ella Ferguson, Odai Mansour, Nicole Brown, Jacob B Stocks, Anja Washington, Victoria Mobley, Shannon Dowler, Jessie Edwards, Lisa B Hightow-Weidman, Christopher B Hurt, Brian Pence, Kathryn E Muessig

**Affiliations:** 1 Department of Medicine University of North Carolina at Chapel Hill Chapel Hill, NC United States; 2 Institute of Global Health and Infectious Diseases University of North Carolina at Chapel Hill Chapel Hill, NC United States; 3 Institute on Digital Health and Innovation College of Nursing Florida State University Tallahassee, FL United States; 4 Department of Health and Human Services North Carolina Division of Public Health Raleigh, NC United States; 5 Department of Epidemiology Gillings School of Global Public Health Chapel Hill, NC United States

**Keywords:** pre-exposure prophylaxis navigation, PrEP navigation, digital health app, mobile health, mHealth, telehealth, public health, sexually transmitted infection, HIV prevention, mobile phone

## Abstract

**Background:**

While access to pre-exposure prophylaxis (PrEP) is an important tool for reducing HIV incidence in the United States, disparities in uptake by race, sex, socioeconomic status, and geography persist. In 2018, the US South accounted for more than half of all new HIV diagnoses but only one-third of PrEP users. PrEP use in North Carolina (NC) similarly lags, with uptake being the lowest among young, sexual and gender minority populations, who account for nearly two-thirds of the state’s incident infections. The PrEP-to-need ratio, a metric of PrEP equity that measures PrEP uptake relative to new HIV diagnoses, highlights disparities in PrEP uptake among specific demographic groups such as women and Black, Hispanic, and Southern people, indicating that these groups are underserved relative to their epidemic need. Despite behavioral risk overlap of incident sexually transmitted infections (STIs) and HIV, in NC, PrEP is only offered at a few primarily urban health department–affiliated STI clinics. The lack of robust health care infrastructure in these areas presents challenges for HIV prevention services.

**Objective:**

This protocol describes a randomized controlled trial of a multilevel PrEP intervention recruiting from rural and periurban STI clinics.

**Methods:**

This trial aims to enroll up to 336 participants and randomly assign them 1:1 to either the intervention or control group. The intervention consists of access to a digital health app, linkage to a remote PrEP navigator, and the option of referral to telehealth-based PrEP services. Persons randomly assigned to the control condition will receive an enhanced standard of care, including access to a limited version of the digital health app. All participants will be followed up on quarterly for at least 3 months. The primary outcome is the initiation of PrEP within 3 months of an index STI clinic visit; secondary outcomes evaluate PrEP care engagement and adherence, incident HIV and bacterial STI infections, PrEP stigma, and cost-effectiveness. Binary outcome analyses will estimate the proportion of participants achieving an event (eg, PrEP uptake) in each arm and a probability difference and the corresponding 95% CI to compare the intervention versus control arm at each time point. Continuous end points will use nonparametric Wilcoxon rank sum tests comparing the intervention and control groups.

**Results:**

Enrollment opened on August 31, 2023, at 15 health departments in NC and subsequently expanded to 21 facilities in 20 counties by July 2024. Completion of the enrollment and data collection phases is expected by May 2025. Results will be published thereafter.

**Conclusions:**

This study directly addresses multiple barriers to PrEP use in rural and periurban areas of the Southeastern United States and can inform policy and programming that seek to expand PrEP access and promote use in underserved communities.

**Trial Registration:**

ClinicalTrials.gov NCT05984030; https://clinicaltrials.gov/study/NCT05984030

**International Registered Report Identifier (IRRID):**

DERR1-10.2196/68085

## Introduction

### Background

To meaningfully reduce HIV incidence, the United States needs integrated, scalable, and cost-effective prevention strategies. Despite the high efficacy of HIV pre-exposure prophylaxis (PrEP), only approximately one-third of PrEP-eligible people nationwide have received a prescription for PrEP; regional and racial disparities have been well characterized and mirror similar disparities in HIV care [1-5]. In 2021, the US South accounted for 52% of new HIV diagnoses but only 38% of PrEP users [6-8]. Coverage of those assigned male sex at birth with PrEP indications was reported by the Centers for Disease Control and Prevention (CDC) as 41% in 2022 but only 12.8% among Black and African American individuals [5]. In North Carolina (NC), the state-level PrEP coverage estimate is 30%, which belies the wide variability from urban to rural areas [9]. Indeed, in NC, where an estimated 1 in 112 residents will acquire HIV in their lifetime [10,11], PrEP use remains below the US average [6,11]. Similar to other states in the Southern region, PrEP uptake is lowest among young, sexual and gender minority men, who account for 76% of new HIV infections in NC [12]. Among the 15 NC counties with the highest rate of HIV diagnoses [13-23], 6 have nonmetropolitan designation, and 8 are small or medium metropolitan areas [12,24]. The lack of a robust health care infrastructure, including scarce HIV care providers, in these areas presents challenges for the sustainable expansion of HIV prevention services.

Similarly to most of the United States, NC’s HIV statistics track alongside those of sexually transmitted infections (STIs) [13], with a disproportionately high burden among rural, young, sexual and gender minority men. Previous studies have demonstrated the potential role of STI clinics as effective PrEP linkage sites but have been limited to urban settings [14-23,25]. Despite behavioral risk overlap of incident bacterial STIs and HIV, PrEP is only offered at a few primarily urban health department–affiliated STI clinics in NC [26,27]. STI clinics are a logical entry point for PrEP services, but ineffective integration in rural STI clinics reflects heterogeneity in clinic structure and staffing—PrEP services require additional human resources and longitudinal engagement to be effective [28]. Leveraging STI clinics as an on-ramp to PrEP presents a compelling opportunity to capitalize on STI service encounters and address disparities in PrEP access for rural residents [15-17].

Multilevel impediments to PrEP scale-up in rural NC include provider shortages, intersectional stigmas, and lack of PrEP knowledge among providers and patients [29-33]. These challenges are compounded by poverty and incomplete insurance coverage of PrEP and PrEP services [34,35]. Building on collaborations with state and local partners, we will implement a multilevel intervention in partnership with rural county health departments and health care organizations that links PrEP and STI services to address PrEP use barriers while working within clinic operational limits and competing demands on physical and human resources. This intervention combines multiple evidence-based interventions, including PrEP navigation services [36-38], access to a digital health platform (HealthMpowerment) [39-42] that connects users to tailored social and informational support for PrEP initiation and persistence [43,44], and referral to telehealth PrEP services [45-48].

### Research Aims

Our objective is to determine the effectiveness and cost-effectiveness of the proposed PrEP linkage strategy in 2 aims. Our primary effectiveness outcome is PrEP uptake within 3 months of an STI clinic visit, defined as confirmed receipt of PrEP prescription or evidence of detectable tenofovir diphosphate in the blood, evaluated through a randomized controlled trial (RCT). Secondary outcomes evaluate PrEP uptake within 6 months of an STI clinic visit, 3- and 6-month PrEP care engagement and adherence, incident HIV or STI infections, and PrEP stigma.

We will examine implementation outcomes as exploratory outcomes, capturing process indicators, including intervention costs, fidelity, and acceptability, to inform future refinement. We will use decision analytic modeling to determine the cost-effectiveness of the proposed intervention over a range of assumptions and inputs, as well as developing a budget impact analysis. Specifically, intervention effectiveness and prospectively collected cost data will be used to model cost per new PrEP initiation. Budget impact analyses will identify drivers of cost, informing strategy refinement for program staffing and scale-up.

This project is supported via a milestone-based funding mechanism, wherein if predefined effectiveness, implementation, and development milestones are met within the initial phase, we will pursue a second phase, in which we will use intervention mapping to refine the proposed strategy and expand implementation in a nonrandomized fashion with updated cost-effectiveness modeling. The second phase is not described further in this paper as the development and finalization of that protocol are dependent on this trial’s outcomes.

This trial has been registered with ClinicalTrials.gov (NCT05984030), which includes reporting of all relevant items from the World Health Organization Trial Registration Data Set.

## Methods

### Protocol Adaptations

We present our methods according to 2 participant recruitment stages: stage I, which focused exclusively on young, sexual and gender minority men seeking STI services from health department–affiliated STI clinics, and stage II, which reflects an amended protocol with modifications made in response to participant enrollment challenges and feedback from collaborating sites. Changes in stage II of protocol implementation include (1) expanding collaborating sites beyond the initially identified 15 health department–affiliated STI clinics, (2) offering a US $5 incentive for completing the brief screening questionnaire, (3) shortening the follow-up period to 3 months for participants enrolled in the final quarter of the study recruitment period, and (4) expanding eligibility criteria to include women assigned female at birth.

Expanding to women more closely aligns with 2021 CDC recommendations, including discussing PrEP with all persons with an STI in the previous 6 months, and reflects the evolving focus on PrEP for women put forth by the CDC, particularly racial minority women in the Southeastern United States. Among new HIV cases in women in the Southern region of the United States, Black women account for 67% of new HIV infections and 72% of new diagnoses among women reporting heterosexual sex [49]. Increasing PrEP use among cisgender women has the potential to significantly impact the spread of HIV in rural and periurban NC and contributes to a broader public health strategy aimed at reducing new HIV infections across various demographics. To explore the potential for our intervention to expand PrEP care to a broader population in NC, we conducted a change in scope that adapted our eligibility criteria to encompass women.

### Study Design (Stage 1)

**Figure 1:**
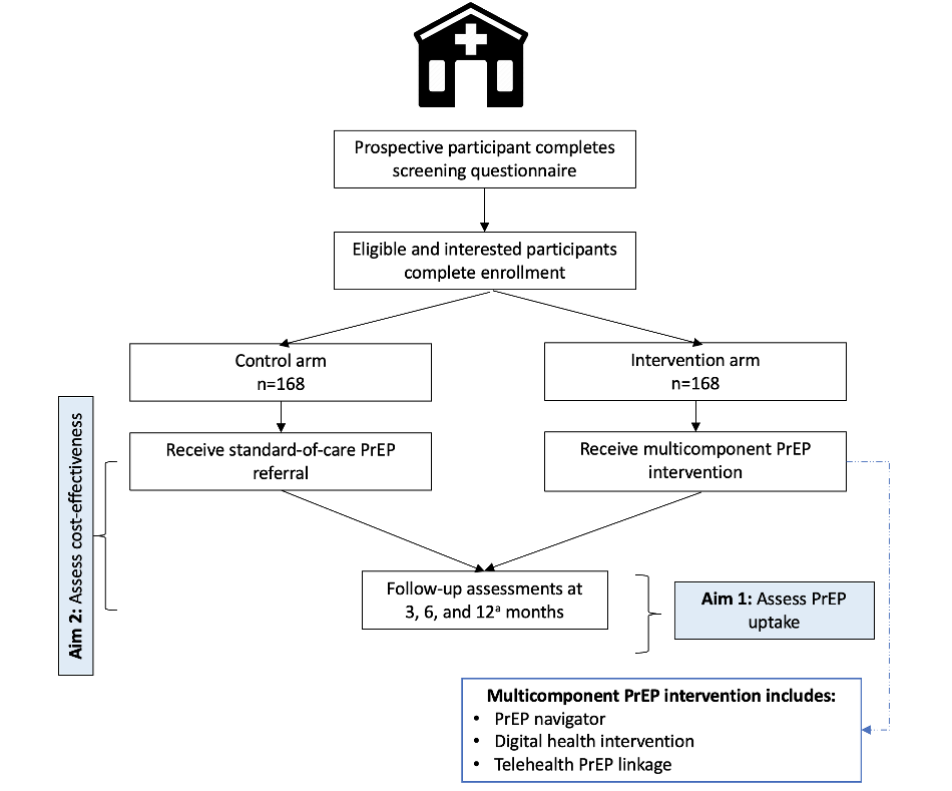
Study schema—aims 1 and 2, years 1 to 3. PrEP: pre-exposure prophylaxis.

This RCT of a multilevel PrEP intervention strategy will open with recruitment of those assigned male sex at birth seeking sexual health services from rural and periurban NC STI clinics (Figure 1). Participants are randomly assigned 1:1 to the intervention or control condition. All participants regardless of study arm complete study measures and activities at enrollment and the 3- and 6-month follow-up time points, with a subset of participants enrolled in the first 6 months of recruitment also completing a 12-month follow-up assessment (Table 1). The timing of participant follow-up activities mimics the CDC-recommended frequency of testing, which includes screening for bacterial STIs every 3 months among men who have sex with men or among patients with ongoing risk behaviors [50,51]. Therefore, HIV and STI testing will be completed as part of patients’ routine care at established STI clinics (not administered through this study). The HealthMpowerment app and study staff will help facilitate participants’ return to the STI clinics for HIV and STI testing by providing in-app appointment reminders, SMS text messages, and phone calls as needed. Quarterly surveys will assess participants’ adherence to CDC-recommended testing frequency.

Cost data and implementation outcomes are collected throughout study implementation examining organization-level determinants of success via surveys and in-depth interviews with participants, clinic staff, health care providers, and other key stakeholders for PrEP expansion.

**Table 1:** Participant assessment timeline.

	Screening	Baseline	3-month follow-up	6-month follow-up	12-month follow-up
Screening questionnaire	✓				
Informed consent		✓			
HIPAA^a^ record release authorization		✓			
App onboarding		✓			
Survey assessment (CASI^b^)		✓	✓^c^	✓	✓^d^
DBS^e^			✓^c^	✓	
Qualitative exit interview (optional)			✓^f^	✓^f^	

^a^HIPAA: Health Insurance Portability and Accountability Act.

^b^CASI: computer-assisted self-interview.

^c^Participants enrolled in approximately the final quarter of study enrollment will only be followed up on for 3 months.

^d^Only applies to participants who enroll during the first 6 months of study recruitment.

^e^DBS: dried blood spot.

^f^A subset of up to 50 participants will complete an in-depth interview following their 3- or 6-month study visit.

### Study Design (Stage 2)

The updated study design incorporates a shortened follow-up period to extend the enrollment window, allowing for a greater number of participants to contribute to the primary outcome of PrEP uptake assessed at 3 months. Randomization and intervention procedures remain unchanged, and study measure assessments are completed at baseline and 3 months (Table 1).

### Eligibility

For stage I, RCT-eligible participants must (1) be assigned male sex at birth, (2) report sexual activity with a male individual in the previous 12 months, (3) report recent HIV testing (within the previous 90 days) and not be known to be HIV positive at screening or enrollment via self-report, (4) be aged 18 to 39 years, (5) have daily smartphone access, (6) be English speaking, and (7) deny recent PrEP use (defined as not having taken oral PrEP or received injectable PrEP within the previous 90 days).

For stage II, the eligibility criteria were updated to reflect the eligibility of persons regardless of sex assigned at birth. All other eligibility criteria from stage I remain unchanged.

We will also conduct qualitative interviews with up to 50 clinic staff members, providers, and other relevant stakeholders for PrEP provision or relevant to the proposed intervention. Interviewees will be purposively sampled to ensure a diversity of clinic roles and responsibilities relevant to STI and sexual health services and referrals, diversity in leadership level, and representation across all participating clinics.

### STI Clinic Selection

We initially identified 15 health department–affiliated STI clinics in relatively high–HIV burden, rural, or periurban counties across NC. Counties were selected in coordination with partners in the NC Department of Health and Human Services using county-level 2019 to 2021 HIV and STI data (reported to the State Laboratory of Public Health). Specifically, we examined HIV and STI testing volume and test positivity rates by reported race or ethnicity among men aged <40 years, prioritizing counties with higher rates of HIV and non-HIV STIs ranked by county without a predefined cutoff. We also reviewed the National Center for Health Statistics urban-rural classification scheme focusing on rural counties, as well as those designated as small (<250,000) or medium (<1 million) metropolitan areas [45-47]. We refer to all locations where STI services are offered as *clinics* or *STI clinics*; however, these locations may also include extensions of clinic services, such as mobile testing or service delivery within other outreach events.

Clinics were contacted with information about the study and an invitation to participate. The study team met with clinics who responded; clinics that decided to participate provided signed letters of support indicating commitment to collaborate and outlining expected roles and responsibilities. At the time of study launch, none of the initial 15 participating STI clinics offered PrEP navigation services or had “in-house” providers prescribing PrEP. Only 7% (1/15) of the participating clinics offered referral to a colocated primary care clinic that could prescribe PrEP. Of the remaining 14 initial collaborating clinics that did not offer in-house PrEP services, a minority provided patients with passive referral to services in the community (eg, the clinic shared the name or names of private practice providers that may offer PrEP services). Starting in March 2024, a total of 6 more clinics were added as collaborating sites under protocol version 3.0. None of these clinics offered PrEP navigation services or had “in-house” providers prescribing PrEP.

### Sample Size and Statistical Power

On the basis of historical clinic volumes and patient demographics, we expect that >7400 patients will present for care across the sites during the study enrollment period, with 20% to 35% being study eligible. Power calculations were based on the study’s primary outcome (PrEP uptake within 3 months of STI clinic visits). PrEP use among the target population has not been estimated previously as there is currently little to no availability of PrEP in rural NC settings. Assuming that 2.5% of eligible patients start PrEP within 3 months in the control arm, we calculated statistical power to detect a range of small to large effect sizes from 5% to 15% increases in PrEP uptake under a range of possible sample sizes and assuming 10% missing data or attrition at 3 months. At a total sample size of 336 given our design and assumptions, we will have >88% statistical power to detect medium (10%) and large (15%) effect sizes and 80% statistical power to detect a moderate (7.5%) effect size with a 5% type-I error rate (SAS; version 9.4 [SAS Institute]).

### Randomization and Enrollment

**Figure 2:**
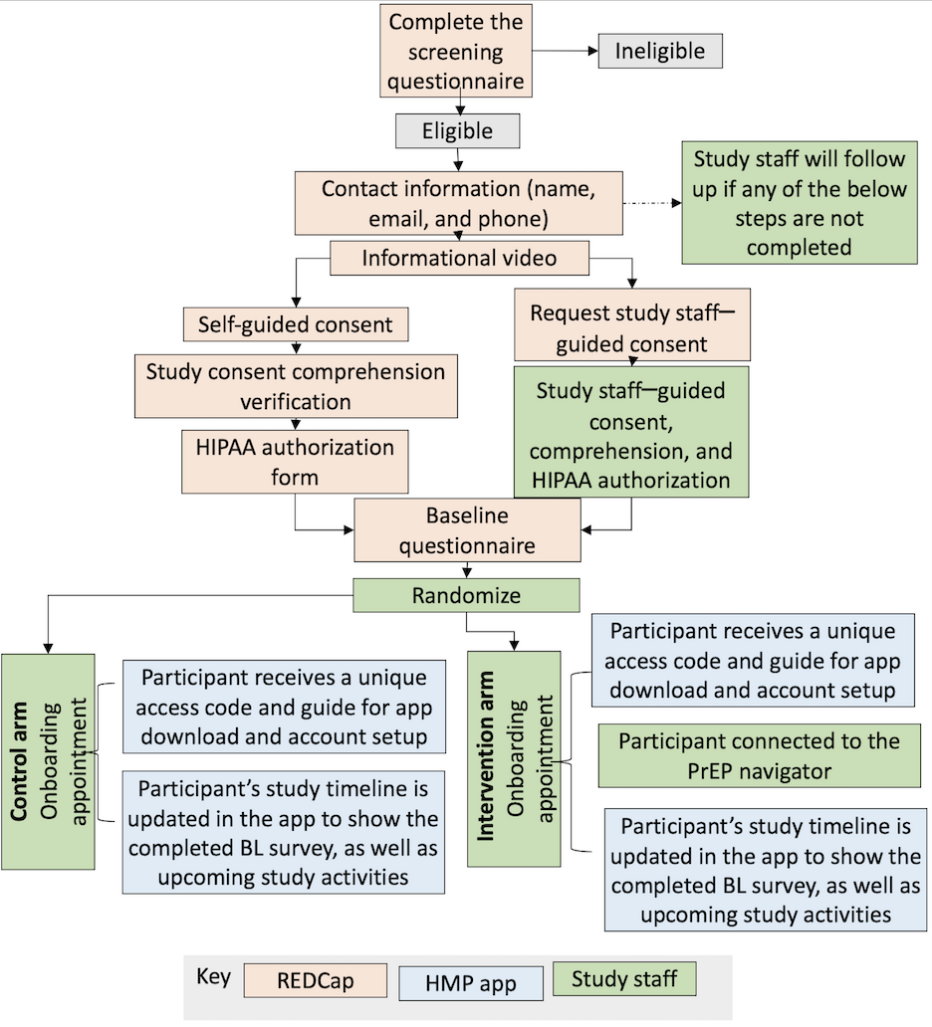
Participant enrollment flow. (Note: Dependent on their enrollment date, only a subset of participants will be asked to complete 6 and 12-month study activities.) BL: baseline; HIPAA: Health Insurance Portability and Accountability Act; HMP: HealthMpowerment; PrEP: pre-exposure prophylaxis; REDCap: Research Electronic Data Capture.

After providing written informed consent electronically and completing baseline surveys, participants will be randomly assigned 1:1 to the control or intervention condition at enrollment using blocked randomization stratified by county, with randomly ordered blocks of sizes 4 and 6 (Figure 2). There will be no masking to assigned arms. Randomization assignments will be automated through the study database. Arm assignment is then disclosed to participants by study staff during study onboarding. The onboarding session includes orientation to study app features for all participants and, for those randomly assigned to the intervention arm, an introduction to the remote PrEP navigator.

### Intervention Arm

#### Overview

**Figure 3:**
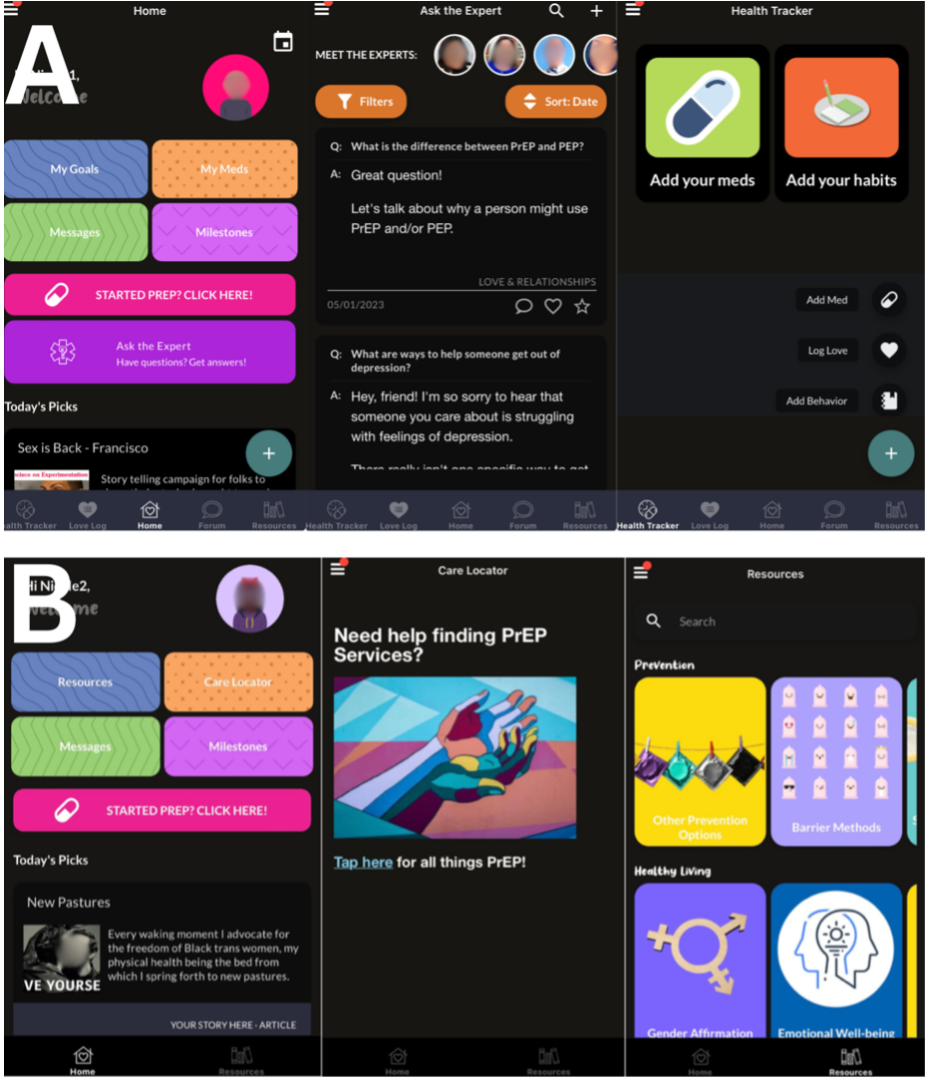
Screenshots from the HealthMpowerment app—(A) enhanced version of the HealthMpowerment app for the intervention condition and (B) limited version of the HealthMpowerment app for the control condition. PEP: postexposure prophylaxis; PrEP: pre-exposure prophylaxis.

Participants randomly assigned to the intervention arm will receive standard PrEP referral services identical to those available to persons in the control condition (see the following sections), as well as a multilevel intervention with 3 components, all of which they engage with remotely: a PrEP navigator to address potential barriers to PrEP uptake and use and facilitate linkage to PrEP services if interested, referral to telehealth PrEP services as an option for linking to PrEP care, and a Health Insurance Portability and Accountability Act (HIPAA)–compliant web-based digital health intervention app (see additional information regarding app privacy in the *Ethical Considerations* and *Data Quality and Monitoring* sections; Figure 3A).

#### PrEP Navigation

PrEP navigators will connect with participants immediately following app onboarding. Navigator services are available to participants for the first 6 months of participation (active intervention period) and include assessing participant awareness of an interest in PrEP, referral to PrEP care, helping participants engage in PrEP care (including assisting with appointment scheduling, reminders, and other provider access issues), and assisting with completing necessary paperwork for insurance and application for drug assistance programs as needed. PrEP navigators undergo study- and navigator-specific trainings.

PrEP navigators were recruited through relevant recruitment channels and listserves commonly used by professionals in the HIV prevention sector. Preference was given to applicants with experience in sexual health; lesbian, gay, bisexual, transgender, and queer health; health insurance; digital health or telehealth; and patient navigation. In addition, candidates with experience collaborating with regulatory bodies and research study teams and in academic-community partnership settings were preferred.

#### PrEP Linkage

PrEP navigators can link interested participants to preexisting local or telehealth PrEP services, although neither are provided, financed, or staffed by the study. Participants referred to telehealth services will receive these PrEP services via the technology platform or service that the provider typically uses. PrEP clinical eligibility, visit frequency, monitoring of laboratory tests, and all other PrEP management will be at the discretion of the PrEP provider. Before enrollment, all participants will sign a HIPAA authorization form permitting study staff to access their medical records pertaining to PrEP care delivery.

#### Digital Health Intervention App

HealthMpowerment is an HIV status–neutral, theory-informed, HIPAA-compliant digital health intervention that supports sexual health and risk reduction among young, sexual and gender minority men [52-54]. HealthMpowerment is guided by the Integrated Behavior Model [55], facilitating examination of the individual and structural determinants of health (eg, stigma and discrimination, health literacy, and poverty) and psychological distress (eg, depression, anxiety, and loneliness) that hinder adoption of HIV-preventive behaviors. The app provides a range of resources addressing these social determinants for persons at all stages of readiness to initiate PrEP, as well as ongoing support and resources for those who do initiate PrEP. All information on the app is available to all participants. The app also recommends specific content to individuals based on their voluntary responses to questions and activities within the app. As part of PrEP-monitoring features, participants can indicate how PrEP was prescribed (ie, daily oral, event-driven oral, or injectable), as well as indicate the dates on which PrEP was taken or, in the case of injectable PrEP, received. HealthMpowerment was first found effective in a statewide RCT [42,53] in NC, which found that greater HealthMpowerment engagement was significantly associated with fewer episodes of condomless anal sex; stigma reduction; and greater provider communication, HIV status disclosure to partners, and HIV care outcomes (eg, engagement in care and adherence) [53,56].

The content included in this version of the app was selected from a broader content library developed and used in previous studies involving similar populations. The app’s design, interface, and features were developed and user tested by a community advisory board comprising racially and ethnically diverse, young, sexual and gender minority groups. In addition, the app’s content is dynamic, with updates made based on user engagement analytics and ongoing developments in the field.

### Control Arm

Persons randomly assigned to the control arm will receive an enhanced standard of care, pairing existing referral systems with access to the core educational resources of the HealthMpowerment app. For this study, access to publicly available PrEP locator resources and tailored information on PrEP and HIV prevention will be accessible from within the app (Figure 3B). This comparator condition of an enhanced standard of care control was selected to balance the dual goals of establishing effectiveness and ethical obligations to provide all participants with information regarding available PrEP services and basic PrEP education.

### Study Procedures

All study staff will be trained in and follow a detailed study-specific procedure manual and topic-specific standard operating procedures (SOPs) that were informed by the Division of AIDS Site Clinical Operations and Research Essentials Manual [57].

### Recruitment

The protocol uses clinic-based recruitment at participating STI clinics. Posters, 1-page flyers, and palm cards with scannable QR codes will be posted throughout the clinic waiting areas, on bulletin boards, and in patient rooms. The study will also be promoted through word of mouth via health professionals, administrative staff, and community outreach personnel associated with each site. Clinic and study staff may also bring recruitment materials to community-based events they attend on behalf of the clinic (eg, health fairs and blood drives) with information about the study.

Scannable QR codes will lead to a web-based screener, which allows potential participants to learn about the study and their eligibility discretely. As of July 17, 2024, prospective participants that complete the screening questionnaire are offered a US $5 e–gift card. Individuals who are not eligible based on screening criteria, duplicate entries, or choosing not to proceed will be thanked and rerouted to a public web page. Prospective participants will be asked to grant permission for study staff to contact them and to provide preferred contact information before they are routed to a brief video describing the research basics and specific study objectives.

### Participant Management and Retention

We will collect multiple forms of participant contact information and study-related communication preferences (eg, email and phone or SMS text messaging) as part of study enrollment. The HealthMpowerment app supports participant retention through a timeline that displays upcoming study activities and a 2-way secure messaging feature for participants and study staff. All participants will receive compensation throughout the course of their participation.

### Data Sources and Collection Methods

Data sources and collection methods are described in this section and summarized in Table 2. The study will use electronic data capture tools (REDCap [Research Electronic Data Capture; Vanderbilt University]) hosted at Florida State University for all primary data collection and participant management purposes [58,59]. REDCap is a secure, web-based software platform designed to support data capture, audit, monitoring, and export for research studies. Study screeners, assessments, and in-depth interviews will all be completed remotely via secure HIPAA-compliant platforms for survey administration and phone and videoconferencing.

**Table 2:** Data sources and collection methods.

Data source and collection method	Primary effectiveness	Cost-effectiveness
CASI^a^ surveys—in the clinic or web-based	✓	
Whole blood collection samples—self-collected blood sample	✓	
Qualitative notes and transcripts—interviews and intervention mapping	✓	✓
Digital health intervention paradata—entered into the app by participants, study staff, and PrEP^b^ navigators	✓	
Clinic observations—project receipts and personnel salary		✓
Time-and-motion logs		✓
HIV and STI^c^ test results—electronic health record and data or Lapcorp or Quest or CELR^d^ or participant-provided records	✓	
PrEP care history—electronic health record data or participant-provided records	✓	✓

^a^CASI: computer-assisted self-interview.

^b^PrEP: pre-exposure prophylaxis.

^c^STI: sexually transmitted infection.

^d^CELR: Clinical and Environmental Laboratory Results.

### HIV and STI Result Abstraction From Electronic Health Records

Participants will be encouraged to receive HIV and STI testing at intervals consistent with CDC recommendations (quarterly testing for men who have sex with men and annual testing for sexually active women) [50]. HIV and STI testing will be completed as part of participants’ routine care at the STI clinic site, not administered through the study or by study staff. Collaborating clinics or PrEP providers will be responsible for specimen collection, processing, and handling of indicated treatment and treatment referral as appropriate. Proof of a nonreactive (negative) HIV assay is required for study enrollment, but no prospective testing is required, nor are participants incentivized to complete these tests. Participants who receive an HIV diagnosis while enrolled in the study will be study stopped. Individuals may choose to keep the digital health app installed on their phone. All test results will be extracted from clinical records. Clinics will either provide study staff with direct access to the participant’s HIV and STI results or upload the results to a secure survey via REDCap.

### Specimen Collection

Dried blood spot (DBS) specimens will be used to detect the presence of PrEP metabolites in participants’ blood and will be administered at months 3 and 6 depending on participants’ enrollment date. Participants can choose to have a DBS kit mailed directly to them (at an address of their choice) or opt for the kit to be mailed to their local health department and pick it up in person. Following an illustrated step-by-step instruction card, participants use a single-use lancet to collect blood onto specialized paper cards, and then they return the sample using a preaddressed, prepaid shipping envelope. DBS specimens will be tested for the presence and levels of tenofovir diphosphate and emtricitabine triphosphate to inform PrEP exposure and prevention-effective use as correlated with survey and available medical record data regarding PrEP medication and intended dosing strategy [60,61].

### Study Assessments

Computer-assisted self-interview survey assessments from REDCap will be sent to participants via the HealthMpowerment app at baseline and 3 and 6 months, with a 12-month brief assessment for a subset of participants. Participants enrolled in approximately the final quarter of enrollment will only complete the 3-month survey, reflecting the shorter follow-up period for this group. Survey domains include sociodemographic data, sexual behaviors, PrEP use, self-reported STI and HIV testing outcomes, and perceived or experienced PrEP stigma.

### Clinic Assessments

Understanding the organizational environment from which participants will be recruited is crucial to interpret outcomes and potential for scale-up and sustainability. We will conduct assessments of all participating clinics to evaluate staffing, patient volume, hours of operation, and available services. These assessments will be conducted using a combination of in-person and remote interactions. We will also ask relevant stakeholders about intervention acceptability and organizational factors that may influence PrEP provision and integration of services, including organizational readiness for change, support climate, and intervention value fit [62-64]. We will also monitor relevant policy changes, such as those pertaining to insurance, PrEP coverage, or recommendations for use that may serve as external influences relevant to our observed outcomes.

### Qualitative In-Depth Interviews

Up to 50 participants will complete an in-depth interview following their 3- or 6-month assessment contextualizing their experience with study participation, perceived accessibility of PrEP before and after study enrollment, evaluation of the acceptability of this enhanced PrEP access model (if randomly assigned to the intervention arm), unmet PrEP-related health service needs and barriers, experience initiating PrEP (if relevant), experience using the study-related intervention components (app, PrEP navigator, and telehealth for PrEP), and other topics as raised by participants (Multimedia Appendix 1). Participant interviews will be conducted remotely via phone or videoconference platform. Participants will be purposively sampled to ensure diversity in areas such as PrEP initiation status, recruitment clinic, age, and race.

In addition, we will engage up to 50 other stakeholders, including health department–affiliated clinic staff, health care providers, and other stakeholders relevant for PrEP services, for in-depth interviews. Interview topics may include experiences and challenges with being part of a participating clinic for this study, perceived accessibility of PrEP for the target patient population before and after study implementation, perceived strengths and drawbacks of this PrEP access model, suggested changes to this strategy, and unmet PrEP-related health service needs and barriers among the participant population. Stakeholder interviews will be conducted in person or remotely via phone or videoconference platform after consent by trained research staff using a semistructured interview guide (Multimedia Appendix 2). Each interview will be digitally recorded and transcribed for analysis.

### Cost-Effectiveness

We will build a decision model to estimate the budget impact and compare the cost-effectiveness and population outcomes of our multilevel PrEP intervention to those of the standard of care.

Specifically, costs will be collected prospectively in 2 ways: microcosting and time-and-motion logs. Microcosting involves “direct enumeration” for consumed inputs [65], an ingredient-based approach. We will further quantify resources associated with the development and implementation of our intervention (eg, costs associated with the adaptation of the HealthMpowerment app and personnel training). Following established methods, we will measure non–research-related costs associated with the intervention and control arms to estimate the incremental cost per additional person starting PrEP. Cost data will be available through contractual information with developers, clinic and project receipts, and NC Department of Health and Human Services supply chain partners. We will also extract data from project expenditure and management records, including purchase logs and human resource records. Time-and-motion assessments record how the involved parties (eg, navigators and providers) divide time among PrEP-related tasks, reliably apportioning effort relevant to implementing the intervention.

### Outcomes and Data Analysis

Our primary outcome (PrEP uptake) is assessed through self-reported PrEP use (oral or injection) on a follow-up survey or on the app and verified by at least one of the following: (1) an uploaded photo or image demonstrating a PrEP prescription, (2) any indication of the presence of tenofovir diphosphate or emtricitabine triphosphate in DBS, or (3) staff-abstracted electronic medical record of PrEP prescription issued or prescriber notation of PrEP being initiated (Multimedia Appendix 3).

### Outcome Analysis

Analyses will be conducted for all primary and secondary outcomes using the following general procedures. Basic descriptive statistics will be calculated. Frequency tables will be presented for the categorical variables, and means, SDs, and percentiles (25th, 50th, and 75th) will be provided for the continuous variables. For each binary outcome, we will estimate the probability of achieving the event (eg, PrEP uptake) in each arm and a probability difference and the corresponding 95% CI to compare the intervention versus control arms at each time point (3-, 6-, and 12-month follow-ups). For continuous end points, we will use a nonparametric Wilcoxon rank sum test to compare the intervention and control arms. For count data variables (eg, number of unprotected sex acts), we will use small analysis methods for count data (eg, exact Poisson regression).

To address missing data, we will review the frequency of missing and nonmissing values for all variables at baseline and the 3-, 6-, and 12-month follow-ups. We will conduct missing value analyses to determine whether persons with missing values are systematically different from those without missing values and whether the probability of having missing values differs by arm. If this assessment of the frequency and imbalance of missing data suggests that bias may be introduced, we will use inverse probability of observation weights or multiple imputation to address the missing data. If there is chance imbalance in the measured baseline covariates between those randomly assigned to the intervention and control groups, we will conduct sensitivity analyses applying stabilized inverse probability treatment weights.

The primary outcome of PrEP uptake is measured at the 3-month follow-up. The effectiveness of the intervention will be estimated as the difference in probability of starting PrEP within 3 months of an index STI clinic visit comparing patients randomly assigned to the intervention and control groups.

Secondary effectiveness outcomes include PrEP uptake at 6 months, PrEP care engagement, PrEP use, PrEP adherence [66-68], incident STIs, incident HIV, and PrEP stigma [69,70] (all at both 3 and 6 months). All analyses will follow the specifications described previously.

PrEP adherence at 3 and 6 months will be assessed using PrEP metabolite levels and self-reported 30-day adherence as 2 separate outcomes. We will use standard-of-care quarterly STI and HIV testing results to examine differential rates of incident STIs adjusting for testing frequency given potential increased testing frequency among PrEP initiators [71,72].

Exploratory outcomes will report the effectiveness of the intervention (defined previously) as measured using PrEP uptake at the 3-month follow-up stratified by sex assigned at birth.

We will also examine additional exploratory implementation outcomes, including process indicators to inform intervention implementation, optimization, and scale-up. We will measure acceptability of the implementation strategies for providers and clinic directors, feasibility of the intervention, and intervention satisfaction among patients and providers using validated scales [73-76] for outcome assessment, as well as measuring cost-effectiveness.

To measure the acceptability [73,77,78] of the intervention and explore clinic-level influences on implementation feasibility at each clinic, we will interview providers and clinic directors. We will assess factors such as provider burden of intervention, adequacy and timing of training, supervision or support structure, organizational readiness for change (quantitative scale and qualitative interviews) [62], management support (qualitative interviews), implementation climate (quantitative scale and qualitative interviews) [63], and intervention value fit (quantitative scale and qualitative interviews) [64]. We will use facility audit data, including county population, patient panel size and demographics, number and training level of clinic staff, staff-to-patient ratio, daily patient volume, opening hours, and local access to PrEP to contextualize determinants.

Feasibility measures will assess patient engagement with the intervention, including uptake of elements (ie, linkage to insurance if not durably insured at enrollment, use of telehealth PrEP, and interactions with PrEP navigators). We will examine the percentage of enrolled participants in the intervention arm who are engaged by the PrEP navigator within 2 weeks of enrollment. We will also examine app-specific engagement, including successful app installation, account creation and log-in, total number of log-ins, and time spent on the app.

Measurement of patient satisfaction [74,79,80] with the intervention includes qualitative (in-depth interview data) and quantitative (eg, System Usability Scale [75]) assessments. We will conduct interviews with a subset of patients regarding their experiences with the intervention. This sample will include a mix of persons with variable HealthMpowerment engagement and PrEP uptake. We will ask about satisfaction with clinician interactions as part of STI or PrEP care. As a quantitative assessment, all participants will complete a satisfaction scale at quarterly follow-up visits [81].

Analysis of implementation outcome measures will include qualitative analysis. Transcripts from participants and providers or clinic staff members will be analyzed separately and reviewed for quality. Interview transcripts will be thematically analyzed following a combination of deductive and inductive analytic approaches. An initial codebook will be developed based on a priori concepts driven by the theoretical underpinnings used to develop the interview guide. All textual data will then be read thoroughly to summarize first impressions. Emerging themes will be incorporated into the codebook. Preexisting codes may be modified based on interview transcripts. Transcripts will be coded iteratively using qualitative analysis software. In total, 2 researchers will code the interviews separately to assess intercoder reliability. The codebook will be revised and updated. Analysis of the coded data will include investigation of relationships between codes, coding matrices, and mapping of codes and themes. Data from qualitative interviews will be triangulated with the quantitative data to gain a more complete understanding of the factors underlying implementation. For example, if our survey data show that clinics with lower fidelity tended to score low on the implementation climate scale, we will examine our qualitative data for indications of what aspects of the leadership or climate may have contributed.

Our primary cost-effectiveness outcome is the incremental cost per additional person started on PrEP (the primary study outcome) in the intervention versus control condition.

We will conduct a budget impact analysis of the cost in US dollars of the intervention overall, providing decision makers with estimates of the financial feasibility of the intervention [82,83].

All models will include sensitivity analyses to examine the potential impact of varying cost and effectiveness assumptions, accounting for parameter uncertainty [84] and capturing input uncertainty (eg, PrEP adherence and intervention cost and effectiveness). The upper and lower bounds will be based on trial data, literature, or expert opinion. The results represent an average across simulated model runs with an estimated uncertainty range.

We will report incremental cost-effectiveness ratios. The analyses will take the perspective of the health system and public payers.

### Study Monitoring and Adverse Event Reporting

The purpose of study monitoring is to verify that the rights and well-being of human participants are protected; the reported trial data are accurate, complete, and verifiable from source documents; and the trial is conducted in compliance with the currently approved protocol and amendments, with good clinical practice, and with all applicable regulatory requirements. This study will follow a detailed clinical quality monitoring plan that specifies the frequency and types of data that will be reviewed (case report forms, regulatory documents, study staff training records, and medical and laboratory records) to accomplish these monitoring activities. External monitoring for this study will be conducted biannually through the North Carolina Translational and Clinical Sciences Institute monitoring service in accordance with established International Conference of Harmonization Good Clinical Practice Guidelines and Title 21 of the Code of Federal Regulations. This review includes quality assurance (QA) and study monitoring, such as regulatory file review, informed consent review, patient eligibility confirmation, protocol compliance review, assessment of safety reporting requirements, and review of training records.

In addition, a study monitoring committee (SMC) will be constituted before initiation of the study. This committee will include, at a minimum, 2 HIV clinicians or research investigators not directly involved in the study and a community representative. At least one representative from the National Institutes of Health (NIH; study sponsor or funder) will be invited to attend the biannual meetings. SMC members will review cumulative study reports, including reports of adverse events (AEs), social harms, and unanticipated problems. The SMC will assess study conduct, adequacy of the delivery of the intervention package, ascertainment of PrEP uptake outcomes, and other related data to ensure adequate collection of primary and key secondary outcome data.

There are no plans for additional interim analyses beyond standard monitoring and reporting, and there are no explicit, prespecified stopping guidelines for the trial. The study sponsor and funder retain the right to terminate the trial.

As a behavioral intervention that does not include an investigational product, standard AE reporting will not be undertaken for this study. The study team will monitor for and track serious and nonserious AEs related or possibly related to study procedures or participation in the study. Study participants will be instructed on how to contact the study staff to report any AEs they may experience at any time between enrollment and the follow-up assessments. AEs will also be actively assessed in all follow-up quarterly assessments. Should a participant report experiencing an AE that they perceive to be related to their study participation, research staff will contact the participant to assess the severity and appropriate resolution action.

### Ethical Considerations

This study, including the protocol, the informed consent documents, and all participant-facing materials, has been reviewed and approved by the University of North Carolina (UNC) Biomedical Institutional Review Board (IRB; 22-3058), who is responsible for the oversight of the study. Any subsequent modifications will be reviewed and approved by the UNC IRB before implementation. Annual IRB reporting and review is required for the duration of the study.

Eligible participants will have the choice to complete a self-guided electronic informed consent form following the informational video before enrollment or complete a staff-guided electronic informed consent form (via a HIPAA-compliant videoconferencing platform or phone call) before enrollment (Multimedia Appendix 4). All participants will be required to complete comprehension questions to ensure that they adequately understand the research, risks, and benefits and that they can opt out of study participation at any time before providing an electronic signature. All prospective participants who provide informed consent will be required to review and digitally sign a HIPAA release form (Multimedia Appendix 5).

All participants will be assigned a unique participant ID number. Participant-related study information will be identified only through the participant ID on all participant case report forms, audio files, transcripts, and computer-assisted self-interview files. Participant names and other personally identifying information will not be used on any study documents and will be redacted from interview transcripts.

Participants will receive digital cash incentives through the Tango payout platform for completing study visits (surveys, DBS kits, and interviews) as follows: US $60 for enrollment activities (baseline survey and enrollment), US $40 for the 3-month survey, US $50 for the 3-month whole blood self-collection kit, US $40 for the 6-month survey, US $50 for the 6-month whole blood self-collection kit, US $40 for the 12-month survey, and US $50 for the qualitative exit interview. Participants can choose their preferred gift card from a reward catalog.

We will secure study data with all appropriate physical, electronic, and operational protections following our data collection and handling SOP. All data files will have encryption and strong password protection, and files will be stored and managed using HIPAA-compliant, secure servers and cloud-based participant management software. Participant names and their participant IDs will be stored in separate tabs in REDCap accessible only to designated study staff and site monitors. Any files that are not specific to a single participant (eg, laboratory sample manifest) will be stored securely on a university-managed, HIPAA-compliant server. Original source documents for individual participants will be maintained at the UNC study site and will be accessible only to the study staff.

### Data Quality and Monitoring

The clinical quality monitoring plan outlines the regular quality control (QC) and QA checks that are completed by the study team and external study monitor. Internal quality management activities ensure that research staff perform the study-required work correctly per the protocol requirements and in real time. Quality management activities also support good clinical practice compliance, human participant protection, and adherence to protocol requirements and site SOPs. Internal QC and QA monitoring and reporting consists of standardized methods and tools for QA and QC checks, as well as mechanisms for summarizing and disseminating the gathered information. Quality management checks will be implemented throughout the data collection process to quickly identify and rectify potential problems. Survey instruments will use skip patterns and built-in checks to minimize discrepant and unrealistic answers. Standard data-cleaning procedures will be used before analyses, including outlier detection and graphical representation of the data.

### Data Dissemination

The results of this research will be disseminated via publication in peer-reviewed journals; conference presentations; and direct presentation to other interested stakeholders involved in HIV prevention and STI care provision, programming, and policy generation. The protocol is available for public review at ClinicalTrials.gov (NCT05984030).

Written publication guidelines for authorship eligibility will follow the criteria recommended by the International Committee of Medical Journal Editors (substantial contribution, participation in writing, approval of final version, and accountability) [85].

Lay summaries and infographics of study findings and implications will also be created for a general public audience and distributed via social media channels, as well as to participating clinics. When participants are asked for consent for the study to maintain their preferred contact information, they are informed that this information may be used to inform them of the study results. Thus, primary results and lay summaries will be individually shared back with participants unless they opt out of receiving this communication.

No participant names or other identifying information will be used in any dissemination materials, published or otherwise. The final deidentified dataset will be maintained by the study primary investigators and may be shared with other investigators for the completion of secondary data analysis following the establishment of acceptable data-sharing or data use agreements per the requirements of the US NIH (funder or sponsor) and UNC at Chapel Hill (grantee).

## Results

Enrollment opened on August 31, 2023, at 15 health department–affiliated clinics in NC and subsequently expanded to 21 facilities in 20 counties by July 2024. Completion of the enrollment and data collection phase is expected by May 2025. As of January 2, 2024, a total of 17 persons have been enrolled. We have conducted 13 PrEP trainings, reaching a total of 106 providers and clinic staff members, as well as organizing 15 refresher trainings to promote continued understanding and study engagement among clinics. We have conducted 33 qualitative interviews among study participants, clinicians, and stakeholders. In addition to the customized HealthMpowerment study app, we have developed a PrEP navigation manual that also includes a compiled list of county-specific resources. The conclusion of phase I of this study is expected by May 2025, with results to be published thereafter.

## Discussion

### Novel Strategies to Address Barriers to PrEP Uptake

This study hypothesizes that leveraging STI clinics as an on-ramp to PrEP will serve as an effective opportunity to capitalize on STI service encounters and address disparities in PrEP access across rural and periurban environments in NC. As infrastructure for providing PrEP in rural NC STI clinics is currently extremely limited, no previous published studies have assessed PrEP uptake in these settings. In some settings, the diverse needs of potential PrEP recipients may exceed what can be reasonably provided by rural STI clinic staff in stand-alone, frequently understaffed clinics. Developing and testing a pathway to PrEP that complements clinic resources and workflow, including linking to remote PrEP navigation and offering high-quality STI testing services or monitoring for PrEP users, is an appealing strategy that could leverage rural STI clinics as a cost-effective on-ramp to sustained PrEP services. We believe that hybridizing components of multiple-effect PrEP uptake strategies, such as access to PrEP navigation and implementation of a virtual PrEP strategy supported by digital health interventions such as HealthMpowerment, may help support PrEP initiation in patient populations that would otherwise face barriers to obtaining and pursuing in-person PrEP service referrals [36,37].

In NC, men assigned male sex at birth who have sex with other men continue to represent a disproportionate majority of new HIV acquisitions. However, as we expanded within rural NC communities and engaged with the rural providers who staff the participating clinics, we recognized the disconnect between this focused recruitment strategy among persons seeking services at STI clinics and the importance of discussing HIV prevention, and PrEP specifically, with all patients. Our expansion to women will offer unique insights into the challenges and opportunities of engaging with this population within rural STI clinics.

Importantly, in this phase of the study, we are screening persons into a research study, not a PrEP referral process or pathway. As such, challenges with the implementation of the study, including recruitment, do not necessarily reflect barriers to linkage to remote navigation and digital health applications that would be implemented were this model to be scaled. Furthermore, although the randomized nature of our approach offers a more robust evaluation of potential efficacy, this design could influence willingness to enroll or engage with study materials. However, the process of referring all persons seeking STI services to PrEP navigation services using a scannable code or offering access to an informational app for those interested, as reflected in the study recruitment process, may indeed be a strategy used in future implementations if our intervention is shown to be effective in improving uptake of PrEP.

### Potential Limitations and Future Directions

The external validity of the intervention may be challenging to establish when implemented outside the context of the research study. Participants are provided with a modest monetary incentive for initial engagement, which could influence their motivation to take part initially. In addition, the provision of ongoing incentives for study activities may impact their continued willingness to participate. This potential limitation will be further investigated through qualitative research aimed at exploring the motivation behind participants’ decision to engage in the study. Importantly, this study was designed explicitly to address challenges related to PrEP access for persons accessing STI services in rural areas in which PrEP providers and general PrEP services are scarce, and thus, the generalizability, including the acceptability of this approach, may not be immediately relevant to other contexts with more concentrated service delivery options and more widely available, robust PrEP referral and linkage opportunities. The potential expansion or modification of this approach to other similarly resource-constrained settings within and outside the United States will be an important next step but is beyond the scope of this pilot. Incorporating PrEP services into STI clinics presents unique implementation challenges. Barriers at the provider (eg, knowledge and self-efficacy), clinic (eg, budget constraints and understaffing), and structural (eg, limitations in capability for long-term follow-up) levels complicate PrEP scale-up in rural STI clinic settings [86]. However, per CDC recommendations, screening for bacterial STIs should occur at least every 6 months for all sexually active patients and every 3 months among men who have sex with men or persons with ongoing risk behaviors [50,51]. By including budget impact analyses, this study informs program planning and sustainability of the proposed intervention strategy. As public health agencies and providers endeavor to increase PrEP uptake, understanding of the comparative value of alternative strategies, particularly among populations with high HIV incidence and low PrEP use, is urgently needed. Successful implementation and scale-up of the proposed intervention relies on understanding the main drivers of clinic-level costs and demonstrating that an integrated strategy for PrEP and STIs may be more cost-effective than providing these services separately, particularly if optimized models of centralized resources (eg, PrEP navigation and telehealth) and local services (eg, regular HIV and STI testing) can be identified. Ultimately, this study will assess the effectiveness, acceptability, feasibility, and cost-effectiveness of a model to colocate STI services and PrEP access in rural and periurban settings and provide critical information about ways to tailor this service delivery model. A pilot study to assess feasibility was not conducted before the full-scale RCT, as the R61 mechanism of the NIH is designed to be informative. This study builds on existing preliminary research that supports the use of PrEP navigators, remote provision of PrEP delivery, and DBS monitoring and draws from extensive literature examining the challenges of HIV care among this population. Further directions of this work will include engaging with state and local stakeholders to refine the PrEP intervention and expanding the refined implementation strategy to all persons who enroll at a participating clinic.

## Appendix

Multimedia Appendix 1 Patient in-depth interview guide.

Multimedia Appendix 2 Clinic stakeholder consent form.

Multimedia Appendix 3 Primary and secondary study outcomes.

Multimedia Appendix 4 Informed consent form.

Multimedia Appendix 5 Health Insurance Portability and Accountability Act authorization form.

## Abbreviations

AE: adverse event
CDC: Centers for Disease Control and Prevention
DBS: dried blood spot
HIPAA: Health Insurance Portability and Accountability Act
IRB: Institutional Review Board
NC: North Carolina
NIH: National Institutes of Health
PrEP: pre-exposure prophylaxis
QA: quality assurance
QC: quality control
RCT: randomized controlled trial
REDCap: Research Electronic Data Capture
SMC: study monitoring committee
SOP: standard operating procedure
STI: sexually transmitted infection
UNC: University of North Carolina

## Data Availability

The datasets generated and analyzed during this study are available from the corresponding author on reasonable request.
