# Supplementary material for: Multilevel Intervention to Support Tailored and Responsive HIV Pre-Exposure Prophylaxis Care in Rural North Carolina: Protocol for a Randomized Controlled Trial
Source: JMIR Res Protoc. 2025 Mar 21;14:e68085. doi: 10.2196/68085 (PMC11971580; doi:10.2196/68085)
Supplement: Multimedia Appendix 1 [file resprot_v14i1e68085_app1.pdf]

Study Title: Meet me where I am: A multilevel strategy to increase PrEP uptake and persistence among young sexual and gender minority men in rural NC (“STARR-NC”)

PHASE 1 Study

Document: Semi-structured Qualitative Interview Guide

Version #: 1.0

Version Date: 2/17/2023

Description of research activity (as provided in the protocol): (n=45 qual interviews)

A sub-set of up to 50 intervention arm participants will complete an in-depth interview following their 3 or 6-month study visit to provide contextualized data about: experience with the study participation, perceived accessibility of PrEP before and after study enrollment, evaluation of the acceptability of this PrEP-access model, unmet PrEP-related health service needs/barriers, experience initiating PrEP (if relevant), experience using the study-related intervention components (app, PrEP navigator, telehealth for PrEP) (if relevant), and other emergent topics as raised by participants. IDIs will be conducted by trained research staff experienced in qualitative research. Interviews will be guided by the use of [this] semi-structured interview guide which includes open-ended questions corresponding to each qualitative endpoint. Interviewers will be allowed the flexibility to probe patient responses and pursue discussion diverging from the initial interview questions if it is relevant to the endpoints of interest. Each interview will be digitally recorded and transcribed verbatim to text for analysis.

Directions for the interviewer: These interviews ask participants questions about their: satisfaction with the intervention and its various parts, thoughts about the intervention’s impact on PrEP accessibility and use, and HIV prevention, and experience with how well the intervention components worked for them. We would like to know what they liked and didn’t like, understood and didn’t understand, and any suggestions for improving the intervention and its components. This information will be analyzed with participants’ survey responses and their app use data to understand how the intervention worked. The findings will be published in academic and mainstream publications, shared with community partners, and used to refine the intervention protocol and implementation strategies.

During the interview, the interviewer can walk the participant through the app on their phone and/or on a shared zoom screen to describe each part of the app/intervention feature before asking questions about it. For aspects of the app/intervention that the participant did not use much, questions can be adapted to focus more on how this feature could be more useful, interesting or motivating. Participants who did not use the intervention components/app very much – or who have not used it recently – may have shorter interviews.

Questions may be asked in different order, not all questions must be asked of each participant if not relevant. For example, participants who have not started PrEP or not accessed various components of the intervention, would not answer those questions.

STARR NC Study Participants Interviewer Guide

INTRODUCTION:

Hi, my name is [FIRST NAME] and I use [PRONOUNS]. I am part of the team working with the University of North Carolina on the STARR NC research study that you've been testing these past few months. Thank you for talking with me today to share your thoughts about this study.

Today I want to learn about your experience and your thoughts in your own words. It's your story and you are the expert. I'm here to learn from you. ***There are no right or wrong answers.*** All of your feedback and ideas will be helpful to us in making this intervention better. And, you don't have to worry about offending me – please feel free to speak your mind.

Some of the topics we talk about today may feel uncomfortable, embarrassing, or upsetting to you as they touch on features of the app and the study intervention that are related to sexual health and other personal behaviors and experiences. As a reminder, this is a voluntary research study and you are free to stop participating at any time, including during this interview. As we're going along you can always say "skip" or "pass" for any question that you do not want to answer.

With your permission, we'll also be asking a few questions today about the at-home finger prick blood test that was part of the study.

I will be audio recording this interview. We will transcribe the recording and the research team will read the texts of this and other interviews to better understand your experiences and opinions about this study. All the data we collect will be anonymous.

Do you have any questions for me before we begin?

INTERVIEW QUESTIONS:

<u>Overall experience</u>
To start off, I'd like to ask you a little bit about your overall experience in this study.

1. When you first started the study, what were you hoping to get out of participating?
   a. How well did your experience meet these expectations?
   b. What parts did not meet your expectations?

2. What have been the best parts about being in the study for you?

3. What things about the study would you suggest be changed in the future?

4. What problems or issues did you have with using any part of the study interventions?
   a. Probe for: Technical problems or other app-related problems
   b. Probe for: Difficulty working with or reaching the PrEP navigator
   c. Probe for: Difficulty with at-home blood sample collection
   d. Other issues?

   Thank you for sharing those issues. I'll be asking you a little bit more detail about each of those as we go through the interview.

PrEP experience

It's helpful if you tell me a little bit about your experiences taking PrEP, so to start…

5. Can you tell me about your experience with PrEP before joining this study?
    a. If prior PrEP use: How did taking PrEP while in this study differ from your prior experience with PrEP, if at all?
    b. What was your impression about PrEP before joining the study? How has that changed since joining the study?

6. How did your use of PrEP change over the course of the study?
    a. Probe for: Start using PrEP?
    b. What are your plans about using PrEP after this study ends?

7. If started PrEP:
    a. Can you tell me about how you accessed PrEP services during the study? (probe for: location, type(s) of provider(s), remote vs. in-person, etc.)
    b. What form(s) of PrEP have you taken during the study? (probe for: daily oral, on-demand, injectable)
    c. What challenges did you encounter during the study with using PrEP as prescribed? What did you do when you met these challenges?
    d. What helped you in taking PrEP daily during the study? What additional supports would you want or need to help you take PrEP?
    e. If not already discussed: Can you tell me about how different parts of this study may have helped you with getting and taking PrEP?

8. If did not start PrEP:
    a. How well did the study do in supporting you in learning about PrEP? How could this be improved?
    b. How well did the study do in supporting you in deciding about whether you would like to take PrEP? How could this be improved?
    c. Deciding whether or not to take PrEP is a personal decision and there is no right or wrong decision. If you are comfortable sharing, what things shape your decision not to take PrEP?
    d. What additional supports would you want or need to help you take PrEP?

9. One goal of this study was to make PrEP more familiar and accessible to people, if they would like to use it.
    a. Thinking about your experience with the study overall, how well do you feel the study met this goal for you?
    b. What do you think could be done differently to better reach that goal?

App experience

Next, I'd like to ask about your experience using the STARR NC study app.

10. First, could you talk a little bit about your overall impression of the study app?
    a. What parts of the app did you like most? Like least?
    b. What features were most useful? Least useful?
    c. Did you have any technical problems using the app? (Probe for details)

11. How did you use the app during the study?
    a. What part of the app or features made you keep using it?

b. How did your app use change over your time in the study?

12. Did you tell anyone about the app?
    a. What did you share with them?
    b. How comfortable would you feel using the app in front of family? friends? a romantic partner? at work? in public?

13. Next, I'll ask you about some of the specific features in the app. It may be helpful for you to open the app on your phone to follow along.
    a. **Resource Center:** How useful was the information in the Resource Center? Are there any specific articles or videos that stood out or that you remember? What other kinds of information would you like to see here?
    b. **Forums:** What did you think about the Forum section? How connected did you feel to the other users on the app through the Forums? How could it be changed to help people feel more connected?
    c. **Medication tracker**: Did you use the app's medication tracker?
        i. How easy was it to use the app to track whether or not you took PrEP each day?
        ii. How did using the app impact your medication taking?
        iii. Were you able to see any patterns on days you didn't take your PrEP?
        iv. How did reviewing your medication calendar influence any decisions about PrEP or sex?
        v. What would you recommend to improve this feature?
    d. **Behavior tracker**: What behaviors, if any, did you use the app to track?
        i. How was your experience using that feature?
        ii. What impact did tracking [behavior] have on you, if at all?
        iii. How did reviewing your behavior tracker data influence any decisions about PrEP or sex, if at all?
        iv. What recommendations do you have for improvement?
    e. **Ask and Expert:** Did you utilize the Ask an Expert Q&A features of HMP?
        i. IF NO: Was there anything particular that kept you from using this feature?
        ii. IF YES: How useful was the information you received?
    f. What else do you remember about using the app? What other recommendations do you have for how the app could be improved?

    g. **If participant is flagged as a "low" user and if these questions haven't already been answered above:** Could you talk about what you think most contributed to your not using the app very much? What did you want from the app that you weren't getting? What other kind of support or tool do you think might be better for you in supporting PrEP information or use?

<u>PrEP navigator experience</u>

Next we'll talk about your experience with the PrEP navigator in this study

14. Did you use the PrEP navigator at all while you were in the study? The PrEP navigator is the person [add names] who talked with you via the app or by zoom or phone about different ways to access PrEP and then may have helped connect you to those services.

    If relevant:
15. Overall, how would you describe your experience working with the PrEP navigator?

16. How well was the PrEP navigator able to meet your PrEP-related requests or needs during the study?
17. How would you describe the amount of time and communications that you had with the PrEP navigator (probe for wanted more, less, just right)?
18. What suggestions would you have for any changes we should make to the PrEP navigator's job in this study?
19. How would you see yourself using a PrEP navigator in the future, if at all?
20. If given the option, would you prefer a remote PrEP navigator like we had in this study, an in-person PrEP navigator at a clinic, or a combination? Please describe what you like about this option.

<u>Telehealth PrEP experience</u>

Next we'll talk about your experience with using telehealth PrEP providers in this study

21. Did you use any telehealth PrEP services while you were in the study? Telehealth is when you meet with a doctor or other healthcare provider remotely – like on a computer or phone – rather than going in-person to a physical clinic or office.

    If relevant:
22. Overall, how would you describe your experience working with the PrEP telehealth provider(s) while you were in this study?
23. How well was the PrEP telehealth provider able to meet your PrEP-related requests or needs during the study?
24. What kind of challenges or issues came up for you in accessing PrEP using telehealth, if any?
25. In addition to using telehealth, what kinds of things did you have to do in-person in order to start or stay on PrEP? (probe for: picking up medications; attending a clinic office visit; getting HIV/STI tests)
26. What suggestions would you have for any changes to the telehealth services?
27. Could you describe your ideal way of interacting with PrEP services (probe for interest in fully remote, fully in-person, hybrid)?
28. How would you see yourself using PrEP telehealth services in the future, if at all?

<u>PrEP dried blood spot self-collection kit experience</u>

Next we'll talk about the part of the study where you were asked to collect a small sample of your own blood from your finger tip.

29. How many blood collection kits did you receive during the study?
    a. If none: probe for why
    b. If any: How many blood sample kits did you complete and mail back during the study?
        i. What was your experience like ordering and receiving the sample kit?
        ii. What was your experience like completing the blood sample kit?
        iii. What difficulties did you have, if any?
30. How would you feel about using this kind of a kit as part of your regular PrEP care? As part of a future research study?
31. If relevant: What kept you from completing or mailing back the kit? What would have motivated/helped you to complete the kit?

<u>HIV/STI services at the health department clinic</u>

Now I'd like to ask you about your experiences getting HIV/STI services at the health department clinic.

32. How satisfied are you with the HIV/STI services you have gotten at the health department clinic during this study?
33. Can you describe any PrEP-related services you have used or are aware of at the clinic?
34. What suggestions do you have for improving the HIV/STI testing and treatment services offered by the clinic?
35. How comfortable do you feel talking with a health care provider at the clinic about HIV prevention options, like PrEP? About sexual health in general?
36. How would you compare your experience getting HIV/STI testing or treatment at this clinic with other HIV/STI testing or treatment experiences you have had?

Wrap up

37. Overall, did you feel like the resources in this study – like the app, PrEP navigator, telehealth, were well-designed for you? Why/why not?
38. Would you feel comfortable referring friends or members of your network to use these kinds of resources? [probe for why/why not.
39. What other ideas or feedback do you think we should talk about related to the study resources or the study itself that we haven't talked about yet today?

Those are all the questions I have today. Do you have any questions for me?

Great. Someone from the study team will contact you shortly about sending your gift card.

Thank you again so much for taking part in this study and in this interview. Your experiences and perspectives are very important to us and helpful in expanding access to PrEP and health services in North Carolina.
