# Supplementary material for: Multilevel Intervention to Support Tailored and Responsive HIV Pre-Exposure Prophylaxis Care in Rural North Carolina: Protocol for a Randomized Controlled Trial
Source: JMIR Res Protoc. 2025 Mar 21;14:e68085. doi: 10.2196/68085 (PMC11971580; doi:10.2196/68085)
Supplement: Multimedia Appendix 2 [file resprot_v14i1e68085_app2.pdf]

University of North Carolina at Chapel Hill
Consent to Participate in a Research Study entitled 'Meet me where I am: A multilevel strategy to increase PrEP uptake and persistence in rural NC (STARR-NC)'- Clinic/Research Staff

Consent Form Version Date: V4.0 dated September 30th , 2024
IRB Study # 22-3058
Department Study ID: IGHID 12221
Principal Investigator: Sarah Rutstein, MD, PhD, and Kate Muessig, PhD
Principal Investigator Department: Department of Medicine
Principal Investigator Phone number: (919) 966-2537
Principal Investigator Email Address: sarah_rutstein@med.unc.edu
Funding Source and/or Sponsor: National Institutes of Health (NIH)

CONCISE SUMMARY

Things you should know about:

- Taking part in this research study is voluntary (your choice). You do not have to participate, and you can leave the study at any time. No matter what you decide, any other care that you get at this site will not change.
- The purpose of the study is to test new ways to increase access to effective HIV prevention medication, called Pre-Exposure Prophylaxis (PrEP).
- You may have been involved as a collaborator with the STARR-NC study thus far. If you choose to participate now as a research participant, researchers will ask you to complete one in-depth interview and complete a survey. The interview will last between 60 - 90 minutes and the survey will take approximately 5 to 10 minutes. Once your interview and survey are completed, your participation as a research participant in the study is complete.
- Common risks from this research include possibly feeling uncomfortable answering questions or others finding out information that you report. While it is helpful if you answer as many questions as you can, you do not have to answer any questions that you do not want to answer. The study staff will make every effort to protect your privacy. There are no anticipated rare or significant risks.
- You may not experience any direct benefit from participating in this study. Your participation in this study has the potential to make a positive contribution to society by identifying new ways to increase access to PrEP among people for whom access to PrEP has been more limited in the past.

Researchers are asking you to join this study because you work at a clinic in which the STARR-NC intervention occurred, or you have been involved as a collaborator to the STARR-NC study, or you are involved in provision of PrEP care or policy-making that is relevant to the implementation of this intervention.

The study staff will offer you a signed copy of this form.

1. What is the key information I should know about this study?

This study involves research. Research is not the same as medical care. Research answers scientific questions. These answers can help find new medicines, treatments, vaccines, and even knowledge on how the human body works. This informed consent form tells you about this study. You can ask questions at any time. You can discuss this study with others before deciding to join.

2. Why are researchers doing this study?

Researchers are doing this study to increase access to Pre-Exposure Prophylaxis (PrEP) across North Carolina. HIV Pre-Exposure Prophylaxis (PrEP) medication has been proven to be effective at preventing HIV infection when taken as prescribed. Even though PrEP has been shown to be effective, not all health care settings currently offer PrEP to their patients. Part of the reason for this is that there are not enough health care providers who prescribe PrEP, and some people have difficulty getting the cost of PrEP and PrEP care covered. The purpose of this study is to test new ways to increase access to PrEP for people in North Carolina. The study includes an intervention that will test whether providing access to resources related to PrEP care at clinics that offer sexually transmitted infection (STI) testing will increase the number of people who start using PrEP. As part of the evaluation of that intervention, we are interviewing health care providers and other relevant stakeholders for the provision of HIV prevention and PrEP services. We want to understand the experience of those who took part in helping implement the intervention as well as those who might play a role in a similar intervention in the future.

3. What will I need to do during this study?

Your participation as a research participant will include one qualitative interview facilitated by a trained research staff member. If you give your permission, this interview will be audio recorded. You will also be asked to complete a brief survey.

4. How long will I be in this study?

If you decide to join, you will be in this study for the duration of one in-depth interview which will last for between 60 to 90 minutes. The survey may be completed in between 5 to 10 minutes.

5. How many participants will be in this study?

Up to 50 clinicians and stakeholders will take part in in-depth interviews as part of this study. Providers and stakeholder interview participants will be selected from across the clinic recruitment site partners as well as other relevant groups affiliated with the study (for example, stakeholders from the North Carolina Department of Health and providers from other clinics that offer PrEP services).

6. What possible risks can I expect from participating in this study?

The following is a summary of the known risks of participating. You may experience all, some, or none of these risks. This study is focused on HIV prevention, which may be a difficult or uncomfortable topic at times. There is some risk of feeling uncomfortable, embarrassed, or upset during your interview discussion. You do not have to answer any questions that you do not want to answer. You may stop the interview at any point if you do not wish to continue with participation in the study. It is unlikely you will be at risk of physical harm as a result of study participation.

Though we make every effort to protect your privacy in this study, it is possible that others may learn about the information you report. You do not have to share any information about yourself that you do not want to share. You may leave the study at any time.

You will be informed if the study staff learns of any new risks related to participation in the study.

7. What possible benefits can I expect from participating in this study?

Research is designed to benefit society by gaining new knowledge. You may not experience any direct benefit from participating in this study. However, because of this study, you may have the opportunity to learn more about HIV risk and prevention. Some people may experience a positive psychological or emotional feeling from participating in an interview that provides the space to talk about your experiences and ideas in a judgement-free way. Your participation in this study has the potential to make a positive contribution to society by identifying new ways to increase access to PrEP among people for whom access to PrEP has been more limited in the past.

8. What other choices do I have?

If you choose not to participate in this study:

- You could choose to join another research study
- You may choose to informally share your experiences about the study with study staff or others outside of the context of the study interview
- You may choose to share your experiences with someone else at a clinic site
- You may choose to do nothing

If you would like more information about the risks and benefits of each one of these choices, talk to the study staff. Your decision not to participate will not lead to any penalty, or loss of benefits or rights that you would normally have otherwise.

9. Can I change my mind about participating in this study?

Yes, you can change your mind at any time. Your participation in this study is completely up to you (voluntary). Your decision to leave the study will not lead to any penalty, or loss of benefits or rights that you would normally have otherwise.

10. Can researchers take me off this study early?

Yes, researchers can take you off this study at any time:

- If researchers believe that your continued participation in the study would not be in your best interest.
- If you do not follow the study requirements. This includes but is not limited to presenting a safety risk to research staff, interfering with study conduct, or fraudulent engagement with study activities.
- If the study is stopped or cancelled for any reason

11. What happens at the end of this study?

Your participation in the study will end after you complete the in-depth interview and survey completion. There are no plans for longer-term follow-up related to this study or any of its procedures.

If the intervention is found to be effective, and funding is available, the study team plans to work with each of the participating clinics to expand the intervention during a second, two-year phase of the project that would enroll eligible future clinic clients in an implementation study. You may have an opportunity to participate in those future efforts in the context of your job/role at the clinic or with the research study.

The study team will share the deidentified results from the study with all study participants, clinicians/stakeholders, and leaders in the North Carolina Department of Health.

12. How will researchers protect my personal (private) information (information collected about me for the study)?

Your participation in this study will be kept confidential and private as permitted by law. This includes the information you provide in the interview.

Researchers have plans and procedures in place to protect your personal (private) information. They keep study records in a secure place. They do not use your name in publications or meetings. Instead, they use a code to link your personal (private) information and your study data. The key to the code will be kept separate from your study data (interview recording and transcript, survey response, and demographic information). Only the researchers can match your name to the code if needed. The audio recording of your interview will be transcribed and then the audio file will be deleted/destroyed. The transcript will be identified only with your code number, not your name, and any identifying details in the transcript will be redacted to protect your privacy. Any information collected about you for the study will be kept confidential and will be shared only with your permission, or as required by law. The results of this research may be published in a medical book or journal or be used for teaching purposes. However, your name or identifying information will not be used. We may use de-identified data from this study in future research without additional consent.

There are some groups watching over this study. They want to make sure that researchers and their staff are protecting your rights and keeping you safe. They also want to see if researchers are following the approved study plan. People from these groups may review your records related to the interview and survey. Researchers may also share any information collected for this study ("personal/private information") with the groups that are listed below. But these groups will only use your personal (private) information for legitimate business, public health, research, regulatory, and commercial purposes. It is important for researchers and the other groups to also share this type of information with regulatory authorities (RAs)/entities (REs) so that they can decide if this new intervention is safe and works the way it is supposed to.

These groups watching over this study and the groups your information is shared with, have a duty to keep your information confidential. Some of these groups are:

- The Institutional Review Board (IRB) of UNC-Chapel Hill
- The U.S. National Institutes of Health (NIH)
- The U.S. Office for Human Research Protections (OHRP)
- Study monitors

Researchers have a Certificate of Confidentiality from the U.S. National Institutes of Health. This certificate is a tool to help protect your personal (private) information (information collected about you for the study). Researchers can use this tool to legally refuse to give your information to others. For example, researchers can say "No" to a court that is trying to get information about you. The court system cannot force researchers to talk about you being in the study.

But the courts can make researchers give personal (private) information about you to prevent serious harm to you or others. And researchers have to give your information to people who work at the organizations that are paying for this study or the U.S. FDA when asked. In this case, the information will be used to check or evaluate the study.

Researchers can release personal (private) information about you when you say it is okay. You can tell others about you being in the study. For example, you can allow your boss, insurer, doctor, or others to get study information. Then researchers cannot use this tool to withhold the information. This tool does not prevent you from having access to your own study information.

A description of the clinical trial that is part of this study will be available on https://clinicaltrials.gov, as required by U.S. Law. This website will not include information that can identify you. At most, the website will include a summary of the results. You can search this website at any time.

13. Will I have to pay anything to participate in this study?

You will not be charged for anything that is done for this study.

14. Will I receive any payment for my participation in this study?

You will receive $50 for participation in the study after completing all study activities (interview and survey). You will not receive partial payment if you choose to end your participation before these study activities are completed.

15. Who should I contact if I think that I am hurt because of my participation in this study?

All research involves a chance that something bad might happen to you. If you think you are hurt because of your participation in this study or have questions about an injury, please tell Dr. Sarah Rutstein. You can do it in person at 2156 Bioinformatics, 130 Mason Farm Road, Chapel Hill NC 27599 or, call 919-843-5859. We can help link you to necessary medical care, if needed. If you become ill or are physically injured during the study, you should seek medical care.

Care for such injuries will be billed in the ordinary manner to you or your insurance company. Since this is a research study, payment for any injury resulting from your participation in this study may not be covered by some health insurance plans. Neither the U.S. NIH nor the University of North Carolina at Chapel Hill will be able to reimburse you (pay you back) for treatment expenses. There is no option for money or other forms of compensation through NIH or the University of North Carolina at Chapel Hill. You do not give up any of your legal rights by signing this consent form.

16. What are my rights and who should I contact if I have questions?

You have the right to leave this study at any time and for any reason. Your choice to participate or not participate in this study does not change your professional role or relationship with this site. You will not give up your legal rights by signing this informed consent form. You also have the right to know about any new information from this study or other related studies. This information may affect your health, well-being (welfare), or decision to stay in this study.

If you have questions or concerns about your rights as a research subject, or if you would like to obtain information or offer input, you may contact, anonymously if you wish, UNC Institutional Review Board (IRB) at 919-966-3113 or by email to IRB_subjects@unc.edu.

17. Do I give researchers permission to contact me?

Sometimes researchers may want to contact you to get more information or to clarify information about you for this study. They may also want to contact you to see if you are interested in joining future studies.

____ (Initials) Yes, researchers may contact me.	____ (Initials) No, researchers may not contact me.

18. How do I confirm my decision to be in this study?

My signature below confirms that the study and this form was explained to me and:

- I had the opportunity to read this form or that it was read to me
- I had the opportunity to ask questions
- I had the opportunity to discuss my study participation with others
- I voluntarily decided to participate in this study

____

Participant's Name (print) **Participant's Signature and Date**

19. Please indicate if you consent to be audio recorded during the interview.

Please **add your initials** the line that best matches your choice:

_____ OK to record me during the interview.

_____ Not OK to record me during the interview.
