# Supplementary material for: Multilevel Intervention to Support Tailored and Responsive HIV Pre-Exposure Prophylaxis Care in Rural North Carolina: Protocol for a Randomized Controlled Trial
Source: JMIR Res Protoc. 2025 Mar 21;14:e68085. doi: 10.2196/68085 (PMC11971580; doi:10.2196/68085)
Supplement: Multimedia Appendix 3 [file resprot_v14i1e68085_app3.docx]

| **Table S1: Primary and Secondary Study Outcomes** | | | |
| --- | --- | --- | --- |
| **Name** | **Time Frame** | **Brief Description** | **Data sources** |
| **Primary outcome** | | | |
| PrEP uptake | 3-month follow-up | Verified self-reported PrEP use (first dose, oral or injectable)^a^ | CASI, in-app report, EHR abstraction, self-collected lab sample |
| **Secondary outcomes** | | | |
| PrEP uptake | 6-month follow-up | Verified self-reported PrEP use (first dose, oral or injectable)^a^ | CASI, in-app report, EHR abstraction, self-collected lab sample |
| PrEP care engagement | 3- and 6-month follow-up | Daily oral or event driven PrEP: after PrEP uptake, number and dates of any subsequent PrEP visits or new/refilled PrEP prescriptions  Injectable PrEP: after initial injection, number and dates of any subsequent PrEP visits/injections | CASI, EHR |
| PrEP use | 3- and 6-month follow-up | Number of consecutive months PrEP used, based on date of first and last dose^b^ | CASI, EHR |
| PrEP adherence (self-report)[65–67] | 3- and 6-month follow-up | Daily oral PrEP: reported PrEP use (past 30 days)  Event-driven PrEP: reported PrEP use corresponding to reported sexual activity (past 30 days)  Injectable PrEP: based on date of injections | CASI |
| PrEP adherence (injection history & drug measurement) | 3- and 6-month follow-up | Daily oral PrEP: PrEP concentrations detected at designated study follow-up visits, based on intraerythrocytic TFV-DP collected as DBS;  Injectable PrEP: based on dates of injections | Self-collected lab sample, EHR |
| Incident STI/HIV | 3- and 6-month follow-up | Clinical test result (yes / no / indeterminate / missing) for each STI and HIV that participant is tested for. | CASI; State, clinic, commercial lab results^c^ |
| PrEP Stigma[68,69] | 3- and 6-month follow-up | Self-reported scored PrEP Stigma scale | CASI |

^CASI: Computer-assisted self-interviewing; DBS: Dried Blood Spot; EHR: Electronic Health Record; HIV: Human Immunodeficiency Virus; PrEP: Pre-Exposure Prophylaxis; STI: Sexually Transmitted Infection; TFV-DP: Tenofovir diphosphate^

^a^ Persons who self-report taking any PrEP (oral or injection) during a follow-up survey OR in their app will meet this endpoint if this self-report is verified by at least one of the following: (1) an uploaded photo or image demonstrating a PrEP prescription; OR (2) any indication of the presence of tenofovir diphosphate in DBS; OR (3) staff-abstracted electronic health record of PrEP prescription issued or physician notation of PrEP initiated.

^b^  First and last dose determined based on self-report for oral PrEP and medical record (or self-report if record not available) for injectable PrEP. Respondents endorsing any PrEP dose will have uploaded current PrEP prescription as described in PrEP uptake outcome above.

^c^ State, clinic and commercial labs: We will collect HIV/STI test results from laboratory portals or via secure upload of results from participants or participating clinics’ medical records.
