# Supplementary material for: Multilevel Intervention to Support Tailored and Responsive HIV Pre-Exposure Prophylaxis Care in Rural North Carolina: Protocol for a Randomized Controlled Trial
Source: JMIR Res Protoc. 2025 Mar 21;14:e68085. doi: 10.2196/68085 (PMC11971580; doi:10.2196/68085)
Supplement: Multimedia Appendix 5 [file resprot_v14i1e68085_app5.pdf]

University of North Carolina at Chapel Hill

HIPAA Authorization for Use and Disclosure of Health Information for Research Purposes

---

IRB Study # 22-3058

Title of Study: IGHID 12221 - Meet me where I am: A multilevel strategy to increase PrEP uptake and persistence in rural NC

Principal Investigator: Rutstein, Sarah
Mailing Address for UNC-Chapel Hill Department: 130 Mason Farm Road, CB7030, Bioinformatics Building Chapel Hill, NC 27599

This is a permission called a "HIPAA authorization." It is required by the "Health Insurance Portability and Accountability Act of 1996" (known as "HIPAA") in order for us to get information from your medical records or health insurance records to use in this research study.

---

1. If you sign this HIPAA authorization form, you are giving your permission for the following people or groups to give the researchers certain information about you (described below):

Any health care providers or health care professionals or health plans that have provided health services, treatment, or payment for you such as physicians, clinics, hospitals, home health agencies, diagnostics centers, laboratories, treatment or surgical centers, including but not limited to the UNC Health Care System and its members and affiliates (collectively, "UNCHCS"), health insurance plans, and government health agencies.

2. If you sign this form, this is the health information about you that the people or groups listed in #1 may give to the researchers to use in this research study:

Any information in your medical records that relates to your participation in this research. These records might include information about HIV/AIDS, other communicable diseases, or HIV Pre-Exposure Prophylaxis care.

3. The HIPAA protections that apply to your medical records will not apply to your information when it is in the research study records. Your information in the research study records may also be shared with, used by or seen by collaborating researchers, the sponsor of the research study, the sponsor's representatives, and certain employees of the University of North Carolina at Chapel Hill or other affiliated entities conducting the research, or government agencies (like the

FDA) if needed to oversee the research study. HIPAA rules do not usually apply to those people or groups. If any of these people or groups reviews your research record, they may also need to review portions of your original medical record relevant to the situation. The informed consent document describes the procedures in this research study that will be used to protect your personal information. You can also ask the researchers any questions about what they will do with your personal information and how they will protect your personal information in this research study.

If study participant payments are described in the consent form, payments may be paid through a partner financial institution or its vendor. By participating in this Study, you authorize the disclosure of limited protected health information to a financial institution or its vendor to facilitate such payments or for tax reporting purposes.

4. If this research study creates medical information about you that will go into your medical record, you may not be able to see the research study information in your medical record until the entire research study is over.

5. If you want to participate in this research study, you must sign this HIPAA authorization form to allow the people or groups listed in #1 on this form to give access to the information about you that is listed in #2. If you do not want to sign this HIPAA authorization form, you cannot participate in this research study. However, not signing the authorization form will not change your right to treatment, payment, enrollment or eligibility for medical services outside of this research study.

6. This HIPAA authorization will not stop unless you stop it in writing.

7. You have the right to stop this HIPAA authorization at any time. You must do that in writing. You may give your written stop of this HIPAA authorization directly to Principal Investigator or researcher or you may mail it to the department mailing address listed at the top of this form, or you may give it to one of the researchers in this study and tell the researcher to send it to any person or group the researcher has given a copy of this HIPAA authorization. Stopping this HIPAA authorization will not stop information sharing that has already happened.

8. You will be given a copy of this signed HIPAA authorization.

____
Signature of Research Subject

____
Date

____
Print Name of Research Subject
